# Phospholipase D1 Signaling: Essential Roles in Neural Stem Cell Differentiation

**DOI:** 10.1007/s12031-018-1042-1

**Published:** 2018-02-24

**Authors:** Shin-Young Park, Joong-Soo Han

**Affiliations:** Biomedical Research Institute and Department of Biochemistry and Molecular Biology, College of Medicine, Hanyang University, 222 Wangsimni-ro, Seongdong-gu, Seoul, 04763 Republic of Korea

**Keywords:** Phospholipase D1 (PLD1), Neural stem cells (NSCs), Neuronal differentiation, Neurogenesis

## Abstract

Phospholipase D1 (PLD1) is generally accepted as playing an important role in the regulation of multiple cell functions, such as cell growth, survival, differentiation, membrane trafficking, and cytoskeletal organization. Recent findings suggest that PLD1 also plays an important role in the regulation of neuronal differentiation of neuronal cells. Moreover, PLD1-mediated signaling molecules dynamically regulate the neuronal differentiation of neural stem cells (NSCs). Rho family GTPases and Ca^2+^-dependent signaling, in particular, are closely involved in PLD1-mediated neuronal differentiation of NSCs. Moreover, PLD1 has a significant effect on the neurogenesis of NSCs via the regulation of SHP-1/STAT3 activation. Therefore, PLD1 has now attracted significant attention as an essential neuronal signaling molecule in the nervous system. In the current review, we summarize recent findings on the regulation of PLD1 in neuronal differentiation and discuss the potential role of PLD1 in the neurogenesis of NSCs.

## Introduction

### Overview of NSCs

**Fig. 1:**
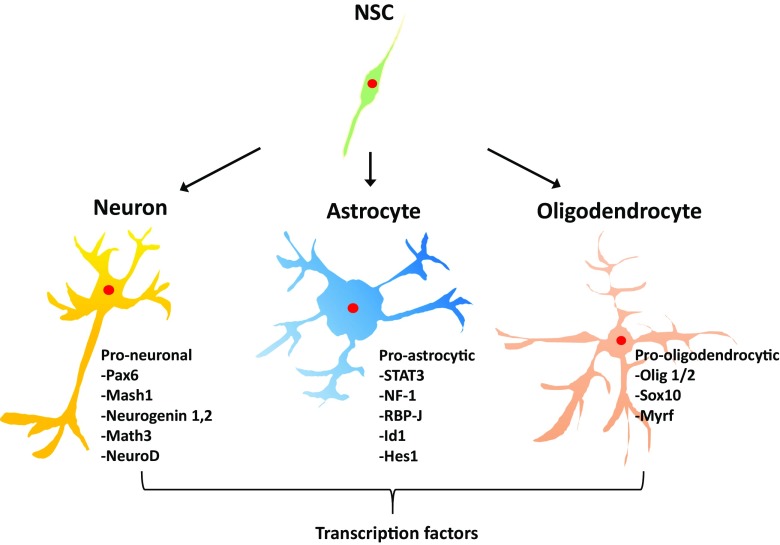
Neural stem cell and major neural cell types. Neural stem cells differentiate into the major neural cell types (i.e., neurons, astrocytes, and oligodendrocytes) depending on their accompanying transcription factors. Pax6, paired box protein; NF-1, nuclear factor-1; RBP-J, recombining binding protein suppressor of hairless; Id1, DNA-binding protein inhibitor 1; Olig1/2, oligodendrocyte-lineage transcription factor; Sox10, SRY-related HMG-box 10; Myrf, myelin regulatory factor

Neural stem cells (NSCs) are multipotent cells that are capable of proliferation and self-renewal, which can differentiate into all types of neural cells, namely neurons, astrocytes, and oligodendrocytes (Miller and Gauthier 2007). In 1992, NSCs were first isolated from the adult striatal tissue, including the subventricular zone and adult mice brain tissue (Reynolds and Weiss 1992). Following the discovery of NSCs, significant advances have been made in our understanding about its localization, development, persistence, properties, and potential in the central nervous system (Xu et al. 2017). Multipotent NSCs can be isolated and cultured from primary cortical or hippocampal cultures after passage in the presence of mitogenic growth factors (Gage et al. 1995), such as epidermal growth factor and basic fibroblast growth factor (bFGF). Mitogenic growth factors are important for NSCs proliferation (Mudo et al. 2009) and the maintenance of its undifferentiated state (Vescovi et al. 1993). The coordinated action of multiple signals acting on embryonic NSCs gives rise to the vast diversity of neuronal and glial populations that populate the mature brain (Xu et al. 2017). Specific transcriptional factors are important for the differentiation of NSCs into the major neural cell types (Fig. 1). NSCs also play a crucial role in animals. In addition to supplying neurons to the olfactory bulb in mice, NSCs are also important for learning and hippocampal plasticity in adult mice (Paspala et al. 2011). Moreover, since the activation of NSCs or their transplantation into areas of central nervous system injury can lead to regeneration in animal models and humans, its putative clinical application has attracted considerable interest.

### PLD Structure

**Fig. 2:**
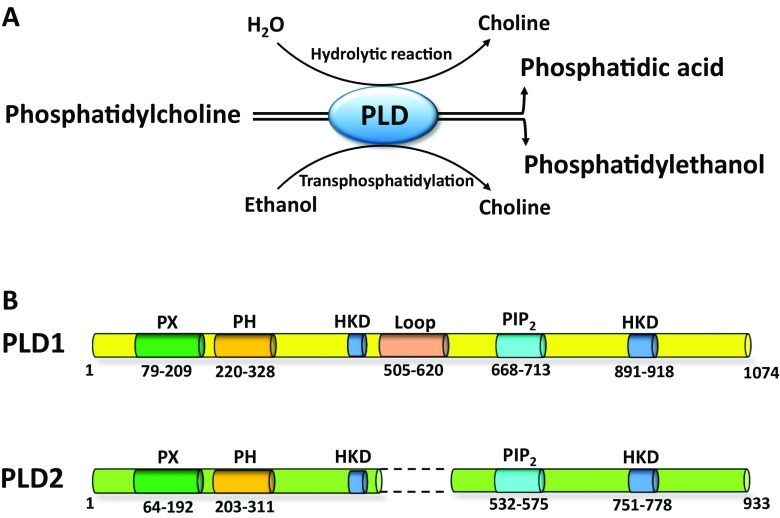
Catalytic reactions of phospholipase D (PLD) and the basic structure of phospholipase D1 (PLD1) and phospholipase D2 (PLD2). **a** PLD hydrolyses phosphatidylcholine (PC) to produce phosphatidic acid (PA) and choline. In the presence of ethanol, PLD preferentially catalyzes the transphosphatidylation reaction rather than the hydrolytic reaction, thus, forming phosphatidylethanol at the expense of PA. **b** Domains shown are the catalytic HKD motif (HKD), phox consensus sequence (PX), pleckstrin homology (PH), phosphatidylinositol bisphosphate (PIP_2_), and PLD1 loop region

Phospholipase D (PLD) is a ubiquitous enzyme that hydrolyzes phosphatidylcholine (PC) to yield phosphatidic acid (PA) and free choline. In the presence of primary alcohols, such as ethanol and 1-butanol, PLD preferentially catalyzes the transphosphatidylation reaction, rather than the hydrolytic reaction, which produces phosphatidyl alcohols at the expense of PA production (Fig. 2a) (Kanaho et al. 2009). Two major PLD isozymes, i.e., PLD1 and PLD2, have been well identified in mammalian cells (Jenkins and Frohman 2005). PLD1 is a 1074-amino acid protein with an apparent molecular weight of 120 kDa. PLD2 is a 933-amino acid protein with an apparent molecular weight of 106 kDa. Mammalian PLD1 and PLD2 both contain two HKD motifs (HxKxxxxD sequence, histidine “H,” any amino acid “x,” lysine “K,” and aspartic acid “D”), which are critical for enzymatic catalysis, both in vitro and in vivo, as evidenced by the observation that point mutations in the motif disrupt PLD activity (Sung et al. 1997). Other highly conserved domains of the PLD isozymes include the phox (PX), pleckstrin homology (PH), and PI4,5P_2_ binding domains, which markedly activates PLD2 and are required for small GTPase ARF stimulation of PLD1 (Exton 2002; Kanaho et al. 2009). Although the PH domain appears to regulate the PLD association with lipid rafts facilitating the recovery of the enzyme to endosomes (Du et al. 2003), it is not required for PLD activity (Sung et al. 1999). The PX domain mediates protein-protein interactions or preferentially binds PI3,4,5P_3_ (Xu et al. 2001). Finally, PLD1 has a conserved loop domain, which is not found PLD2. This loop domain is involved in the auto inhibition of PLD1, since its deletion from PLD1 results in high basal activity (Fig. 2b).

### PLD Functions

Numerous reports suggest that PLD1 contributes to various cellular mechanisms, including inflammation, tumor cell invasion and metastasis, lipid metabolism, and neural development (Bae et al. 2014; Brown et al. 2017; Bruntz et al. 2014). Therefore, PLD1 have emerged as drug targets for many diseases such as infectious diseases, cancer, cardio-vascular diseases, and neurodegenerative diseases (Brown et al. 2017; Eftekharian et al. 2017). PLD1 is found throughout the cell, particularly, in the perinuclear region, Golgi complex, and early endosomes in non-stimulated cells. Further, it is relocated to the plasma membrane upon stimulation. Increased expression of PLD1, its subcellular localization and altered catalytic activity have essential roles in cell proliferation, differentiation, vesicle trafficking, and cytoskeleton rearrangement in neuron (Brito de Souza et al. 2014; Luo et al. 2017). PLD1 is expressed in many functionally diverse brain areas, including the cerebral cortex, hippocampus, brain stem, spinal cord, and olfactory bulb (Lee et al. 2000). Recent studies have reported that the signal-dependent activation of PLD1 is important for neuronal differentiation in NSCs (Park et al. 2015, 2017; Yoon et al. 2005, 2006). PLD2 is almost exclusively found in the light membrane “lipid raft” fraction of the plasma membrane (Gomez-Cambronero and Keire 1998). PLD2 can be activated in intact cells by a variety of agonists and tyrosine kinases. Further, it can be regulated by small GTPases and certain PKC family members (Gomez-Cambronero 2014). PLD2 promotes neurite outgrowth in PC12 cells and functions as a downstream signaling effector of extracellular signal-regulated kinases in the nerve growth factor (NGF) signaling pathway. In PC12 cells and cerebellar granule neurons, this pathway is activated by NGF and neuronal cell adhesion molecule L1 (Watanabe et al. 2004; Yun et al. 2006). Therefore, both PLD1 and PLD2 appear to influence neurite outgrowth. However, the role of PLD2 in neuronal differentiation of NSCs has not yet been elucidated. Therefore, this review focused on the role of PLD1 in neuronal differentiation and described its potential role in the neurogenesis of NSCs.

## Role of PLD1 in Neuronal Differentiation of NSCs

In HiB5 cells, the activation of PLD contributes to neuronal differentiation via neurogenic platelet-derived growth factor (PDGF) (Sung et al. 2001). Further, NGF-induced PLD1 expression mediates neuronal differentiation of PC12 cells (Ammar et al. 2013; Min et al. 2001). PLD1 is also implicated in the bFGF-induced neurite outgrowth of H19-7 cells (Klein 2005; Yoon et al. 2012). In addition, PLD1 corrected the impaired neurite outgrowth capacity of familial Alzheimer’s disease mutant neurons (Cai et al. 2006). Thus, PLD1 is a key molecule in neuronal differentiation, especially neurite outgrowth. Yoon et al. (Yoon et al. 2005) reported for the first time that PLD1 is required for neurite outgrowth during neuronal differentiation of NSCs. Since then, PLD1-mediated signaling pathways have been identified in neuronal differentiation of NSCs. Herein, we summarize the PLD1-mediated signaling molecules involved in the neuronal differentiation of NSCs.

### PLD1 and Rho Family GTPases in Neuronal Differentiation of NSCs

During brain development, each neuron develops into a single axon and multiple neurites, which then eventually form synapses (Elston and Fujita 2014; Huang et al. 2017). To ensure precise neuronal connectivity, neurons are derived from the coordination of multiple developmental steps, including axon growth, branching, guidance, and synapse formation (Huang et al. 2017). Cytoskeleton rearrangement is required for the dynamics of neuronal morphology formation. The Rho family GTPases, of which RhoA, Cdc42, and Rac1 are best characterized, act as significant modulators of cytoskeleton rearrangement (Threadgill et al. 1997). The Rho family GTPases serves as a molecular switch by converting from an inactive GDP-bound state to an active GTP-bound state. Once activated, they can interact with their specific effectors. Recent reports suggest that RhoA, Rac1, and Cdc42 play a central role in dendritic development. Further, the differential activation of Rho-related GTPases contributes to the generation of morphological diversity in the developing cortex (Threadgill et al. 1997). Rac1 and Cdc42 promote neurite initiation and outgrowth (Daniels et al. 1998). Conversely, RhoA activation antagonizes neurite formation and causes neurite retraction. Thus, the regulation of Rho family GTPases is crucial for guiding downstream biological reactions, such as axon growth or retraction, and synapse maturation during neuronal development.

The Rho family GTPases are important regulators of PLD activity (Powner and Wakelam 2002). PLD1 activity is regulated particularly by interactions with small GTPases that belong to the ARF and Rho families (Powner and Wakelam 2002; Rudge and Wakelam 2009). The transfection of RhoA, Cdc42, or Rac1 can activate PLD1 (Powner and Wakelam 2002; Yoon et al. 2006), which has been implicated in the regulation of the actin cytoskeleton (Rudge and Wakelam 2009). PLD1 controls many physiological functions, such as cell migration and neuronal axon formation, via this regulatory action. In NSCs, the expression levels of Cdc42 and RhoA were increased during neuronal differentiation, and PLD1 and Cdc42 were co-localized in neurites, while RhoA was localized in the cytosol (Yoon et al. 2006). Further, Cdc42 was bound to PLD1 during differentiation, and dominant-negative Cdc42 (Cdc42N17) decreased PLD activity and neurite outgrowth. Conversely, constitutively active Cdc42 (Cdc42V12) increased both PLD activity and neurite outgrowth, suggesting that the association between Cdc42 and PLD1 is important for the activation of PLD1 and neurite outgrowth in NSCs. Moreover, a dominant-negative Rac1 (Rac N17) mutant inhibited PLD1-induced Bcl-2 expression. Bcl-2 expression, however, was not altered by DN-Cdc42 (Cdc42 N17) or DN-Rho (Rho V19) during neuronal differentiation of NSCs (Park et al. 2015). Therefore, the interplay between PLD1 and Rho family GTPases has an important role in the neuronal differentiation of NSCs.

### PLD1 and Bcl-2 Expression in Neuronal Differentiation of NSCs

Bcl-2 is a well-known anti-apoptotic protein that prevents the release of apoptogenic factors, such as cytochrome c and second mitochondrial-derived activator of caspase, which was originally found to be overexpressed in B cell lymphoma (Gross et al. 1999). Bcl-2 serves as a critical regulator of pathways involved in apoptosis and inhibits cell death (Liu et al. 2013). Proteins of the Bcl-2 family influence neuronal apoptosis and cell differentiation and a reduction in the ability of neurons to extend neurites in Bcl-2-deficient embryos (Chen et al. 1997; Yoon et al. 2012). Bcl-2 is critical for the neuronal commitment of mouse embryonic stem cells (Trouillas et al. 2008). Moreover, the anti-apoptotic role of Bcl-2 has been well identified in previous studies, in which anti-apoptotic gene modifications have had beneficial effects on the neural differentiation of neural progenitors and NSCs (Esdar et al. 2001; Lee et al. 2009). In vivo studies also indicated that the overexpression of Bcl-2 enhanced retinal axon regeneration after optic-tract transaction (Chen et al. 1997) and increased axonal growth of transplanted fetal dopaminergic neurons in the rat striatum (Holm et al. 2001).

Recent studies have demonstrated that Bcl-2 is implicated in PLD1-mediated neuronal differentiation. PLD1 is known to regulate Bcl-2 expression in various cells (Cho et al. 2008, 2011; Choi and Han 2012). For instance, PLD1 regulates Bcl-2 expression via the JNK/STAT3 pathway, which leads to neuronal cell differentiation of H19-7 cells (Yoon et al. 2012). A recent study also demonstrated that PLD1 increased Bcl-2 expression and promoted Bcl-2-mediated signaling in NSCs (Park et al. 2015). More specifically, PLD1 is regulated by PLCγ/PKCα activation and promotes Bcl-2 expression, via the PA/AA/PGE2/EP4/PKA/p38 MAPK pathway during neuronal differentiation. These results suggest that PLD1-mediated Bcl-2 expression affects the neuronal differentiation of NSCs.

### PLD1 and Ca^2+^-Dependent Signaling in Neuronal Differentiation of NSCs

The development of the nervous system occurs through a series of well-organized steps in the proliferation of NSCs, its migration over considerable distances from the germinal centers to their destinations, and ultimately their differentiation into billions of neurons and glia, which populate the brain (Toth et al. 2016). In these processes, Ca^2+^ signaling is essential for the developing brain (Zheng and Poo 2007). Increased Ca^2+^ levels regulate PKCα activation and translocation to the membrane from the cytosol in various processes (Boncoeur et al. 2013; Champion and Kass 2004). PKCα regulates Ca^2+^-dependent differentiation in several cell lines and primary cells and plays an essential role in synaptic plasticity by raising intracellular Ca^2+^ levels (Kopach et al. 2013; Park et al. 2015). PLD catalyzes the hydrolysis of PC to PA and choline (Exton 2002). PA itself acts as a cellular messenger or is further transformed by PA phosphohydrolase into DAG, which is essential for the activation of PKC (Zhao et al. 2007). The activation and phosphorylation of PLD1 is regulated by PKCα, with a similar interrelationship between PLD and PKC isoforms seen in a variety of cell types (Kim et al. 2005; Park et al. 2015). Recent studies revealed that increased intracellular Ca^2+^ affects PKCα activation and neurite outgrowth in NSCs (Park et al. 2015, 2017). In addition, a PKCα specific inhibitor, RO320432, reduced the activation of PLD1 and affected PLD1 signaling during differentiation in NSCs (Park et al. 2015, 2017). Moreover, intracellular Ca^2+^ promotes neurogenesis by translocating PKCα to the membrane through making complex with hippocalcin (HPCA). And then PKCα is activated by direct binding to phosphoinositide-dependent protein kinase 1 (PDK1) in NSCs. PDK1 signals upstream of PKCα trigger neurite outgrowth leading to increased expressions of *Nt3*, *Nt45*, *Bdnf*, and *Neuro D* in NSCs (Park et al. 2017).

Another important Ca^2+^ signaling factor, phospholipase C (PLCγ), also affects PLD1 signaling in several cells (Park et al. 2009, 2015; Yoon et al. 2012). When treated with some growth factors, PLCγ is phosphorylated and generates DAG and inositol 1,4,5-triphosphate (IP3), which in turn activates PKCα, consequently increasing intracellular Ca^2+^ (Hall et al. 1996; Oh et al. 2008). Recent studies demonstrated that PLCγ signaling elevates the intracellular Ca^2+^ concentration and regulates neocortical neuronal progenitor migration and neuronal differentiation (Lundgren et al. 2012; Park et al. 2015). Moreover, the inhibition of PLCγ using a specific inhibitor, U73122, or blocking intracellular [Ca^2+^]i with BAPTA-AM, reduced the phosphorylation and activation of PKCα during neuronal differentiation of NSCs (Park et al. 2015). Furthermore, U73122 or BAPTA-AM inhibited PLD1 activity and neuronal differentiation in NSCs (Park et al. 2015). Taken together, these results suggest that intracellular Ca^2+^ signal molecules, including PLCγ, PKCα, and PDK1, regulate PLD1-mediated neuronal differentiation in NSCs.

HPCA is a high-affinity Ca^2+^-binding protein, which is restricted to the CNS and most abundant in pyramidal cells of the CA1 region in the hippocampus (Kobayashi et al. 2005). During brain development the expression of HPCA sharply increases concurrently with synapse formation (Saitoh et al. 1994). HPCA belongs to the family of EF-hand-containing neuronal Ca^2+^ sensor proteins, which possess a Ca^2+^/myristoyl switch that allows its translocation to the membrane, in response to increased cytosolic Ca^2+^ concentrations (Oh et al. 2008; Park et al. 2017). HPCA exerts a neuroprotective action by blocking the formation of Ca^2+^-induced cell death stimuli (Masuo et al. 2007). Further, infusion of mutant Hpca lacking Ca^2+^-binding sites prevents long-term depression in hippocampal neurons (Jo et al. 2010). Since HPCA has a crucial role in Ca^2+^-mediated neuronal activity in the brain, it is possible that HPCA is implicated in neuronal differentiation of NSCs. HPCA is also regulated by a Ca^2+^-mediated PLD1 signaling pathway (Oh et al. 2008; Park et al. 2017). It also induces the expression of neuro-D, leading to neurite outgrowth during differentiation in H19-7 cells (Oh et al. 2008). A recent study demonstrated that the expression of nerve growth factors, such as *NT-3*, *NT-45*, and *BDNF*, depended on Ca^2+^ binding and the myristoylation of HPCA during the neuronal differentiation of NSCs (Park et al. 2017). Interestingly, HPCA directly binds to PKCα, which facilitates the PKCα-regulated kinase cascade; PKCα-dependent PLD1 activation is required for neurite outgrowth. Moreover, PLD1 and HPCA were even co-localized on embryonic day 14 (E14) in the rat cerebral neocortex, and HPCA-dependent PLD1 activation was required for neuronal differentiation of NSCs. Finally, their collaboration greatly influenced the neurogenesis of NSCs (Park et al. 2017).

## PLD1 as an Accelerator in Neurogenesis of NSCs

Neurogenesis is the transition of proliferative and multipotent NSCs to fully differentiated neurons. It occurs in multiple brain areas, including the neocortex, piriform cortex, amygdala, substantia nigra, striatum, and hypothalamus (Iannitelli et al. 2017). Neurogenesis is the process by which neurons are generated from neural stem cells and progenitor cells. It precedes gliogenesis throughout the nervous system, and a single progenitor can give rise to both neurons and astrocytes (Bayer et al. 1991). Neurogenesis is tightly controlled owing to its critical importance in proper physiological function, and the multiple signals controlling the growth and directionality of the relevant cell fate decision (Sun et al. 2001). To promote neurogenesis, proneural basic helix-loop-helix (bHLH) transcription factors, such as neurogenin-1 and Mash-1, not only drive neurogenesis by activating the expression of a cascade of neuronal genes (Frohman et al. 1999) but also through inhibiting glial gene expression (Urban and Guillemot 2014). However, some neurogenic factors can regulate both these processes, depending on the concentration of proneural genes. For example, although bone morphogenetic proteins promote neurogenesis in progenitor cells that express high levels of neurogenin-1, it promotes gliogenesis in progenitor cells that have a low level of neurogenin-1 expression (Morrison 2001). Thus, embryonic neurogenesis is tightly linked to cell fate specification. Moreover, according to recent studies, the molecular and genetic factors influencing neurogenesis notably include the Notch pathway; many genes have been linked to Notch pathway regulation (Kageyama et al. 2008; Rash et al. 2011).

### How Does PLD1 Promote Neurogenesis in NSCs?

Over the past year several regulatory mechanisms, including the promotion of neurogenesis by proneural bHLH genes and the instruction of gliogenesis by signal transducers and activators of transcription 3 (STAT3) in a neurogenic capacity of NSCs in culture, have been identified (Kang et al. 2016; Park et al. 2017). STAT3 is an important transcription factor that regulates glial fibrillary acidic protein (GFAP) expression. Further, the DNA binding of STAT3 was affected by the phosphorylation of the Ser727 or/and Tyr 705 site (Yokogami et al. 2000). STAT3 binds to different domains of CBP/p300 and the STAT/p300/Smad complex, acting at the STAT-binding element in the astrocyte-specific GFAP promoter, which is particularly effective at inducing astrocyte differentiation in NSCs (Nakashima et al. 1999). SH2-domain-containing tyrosine phosphatase-1 (SHP-1) negatively regulates STAT3 signaling through the direct de-phosphorylation of STAT3 (Tyr 705). Importantly, this SHP-1-dependent STAT3-inhibitory mechanism is closely involved in PLD1-directed neurogenesis in NSCs. PLD-derived PA interacts with and inhibits SHP-1 activity (Frank et al. 1999). Exogenously added PA induced phosphorylation of SHP-1 and de-phosphorylation of STAT3 (Tyr 705) in a dose-dependent manner in NSCs. Moreover, PLD1 knockdown inhibited SHP-1 activity and affected the de-phosphorylation of STAT3 (Tyr 705). Thus, PLD1 promotes neurogenesis and suppresses gliogenesis by controlling the activation of SHP-1/STAT3 in NSCs. Therefore, PLD1/PA/SHP-1/STAT3 signaling is an important pathway in embryonic brain neurogenesis.

## Conclusions

**Fig. 3:**
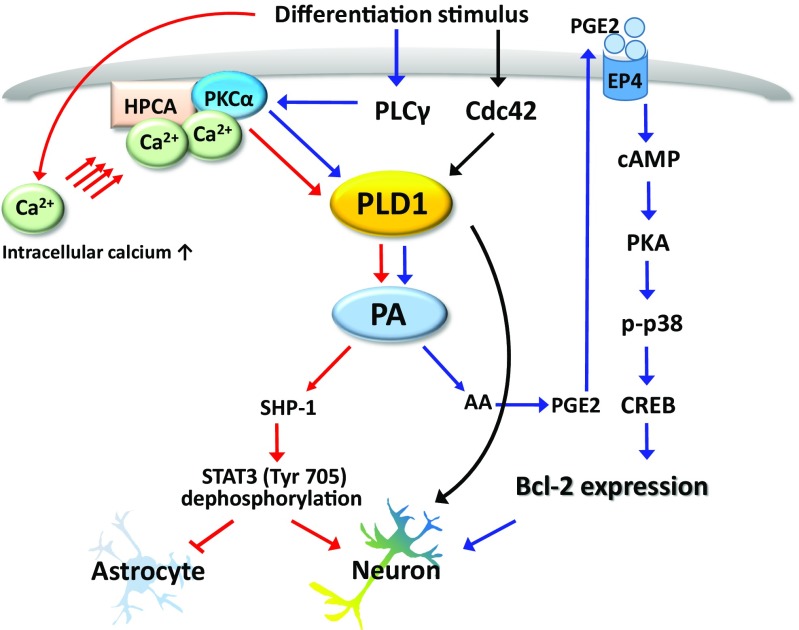
Phospholipase D1 (PLD1)-mediated multiple signals contribute to promote neurogenesis of neural stem cells (NSCs). At least three different signals are involved in the regulation of neuronal differentiation of NSCs. Ca^2+^-dependent signaling (red arrows) is the most important among these signals. Increased intracellular Ca^2+^ induces hippocalcin (HPCA)-protein kinase Cα (PKCα) activation, which facilitates PKCα-dependent PLD1 activation. Phosphatidic acid (PA), a functional product of PLD1, affects the activation of SH2-domain-containing tyrosine phosphatase-1 (SHP-1). SHP-1 inhibited the activation of STAT3 (Tyr 705) activation, thereby inhibiting astrocytic differentiation and promoting neuronal differentiation in NSCs. The second proposed model for pathway signaling is the PLD1-mediated Bcl-2 expression during neuronal differentiation of NSCs (blue arrows). The model suggests that Bcl-2 expression in neuronal differentiation of NSCs, including neurite outgrowth, depends on PLCγ/PKCα/PLD1/PA/AA/EP4/PGE2/PKA/p38MAPK/CREB/Bcl-2 signaling. The final pathway is the binding of Cdc42 to PLD1, which increased PLD1 activity during neuronal differentiation of NSCs (black arrows). PLD1 activation by Cdc42 increased neurite outgrowth, suggesting that PLD1 activity is required for neuronal differentiation in NSCs

To summarize the findings presented thus far, PLD1 is critical for neuronal differentiation, which is regulated by multiple signals, contributing to the neuron-to-astrocyte switch in NSCs from the rat E14 cortex (Fig. 3). Therefore, PLD1 may have a positive role in neuronal differentiation of NSCs. Conversely, however, it has also been reported that PLD1 plays a negative role in neuronal differentiation, especially in the dendritic branching of cultured hippocampal neurons from rat E18 (Zhu et al. 2012). In culture, progenitor cells isolated at different embryonic stages behave in a manner that mimics the normal process of development. Progenitor cells from rat E14 cortex (at the peak of neurogenesis) primarily give rise to neurons and dividing precursor cells. In contrast, E18 progenitor cells immediately give rise to astrocytes (Sun et al. 2001). These studies have demonstrated that the role of PLD1 may be reversed depending on the age and location of the stem cell embryo. In this regard, we should now consider the study of how PLD1 regulates neurogenesis according to the age and location of the embryo. Addressing this will provide us with insights into the differentiation mechanisms of neural stem cells following the developmental stages of the brain. Further, it may also help us in the application of neural stem cells to repair the damaged or degenerative nervous system.
